# Creation and disruption of protein features by alternative splicing - a novel mechanism to modulate function

**DOI:** 10.1186/gb-2005-6-7-r58

**Published:** 2005-06-22

**Authors:** Michael Hiller, Klaus Huse, Matthias Platzer, Rolf Backofen

**Affiliations:** 1 Institute of Computer Science, Friedrich-Schiller-University Jena, Chair for Bioinformatics, Ernst-Abbe-Platz 2, 07743 Jena, Germany; 2 Genome Analysis, Institute of Molecular Biotechnology, Beutenbergstrasse 11, 07745 Jena, Germany

## Abstract

**Background:**

Alternative splicing often occurs in the coding sequence and alters protein structure and function. It is mainly carried out in two ways: by skipping exons that encode a certain protein feature and by introducing a frameshift that changes the downstream protein sequence. These mechanisms are widespread and well investigated.

**Results:**

Here, we propose an additional mechanism of alternative splicing to modulate protein function. This mechanism creates a protein feature by putting together two non-consecutive exons or destroys a feature by inserting an exon in its body. In contrast to other mechanisms, the individual parts of the feature are present in both splice variants but the feature is only functional in the splice form where both parts are merged. We provide evidence for this mechanism by performing a genome-wide search with four protein features: transmembrane helices, phosphorylation and glycosylation sites, and Pfam domains.

**Conclusion:**

We describe a novel type of event that creates or removes a protein feature by alternative splicing. Current data suggest that these events are rare. Besides the four features investigated here, this mechanism is conceivable for many other protein features, especially for small linear protein motifs. It is important for the characterization of functional differences of two splice forms and should be considered in genome-wide annotation efforts. Furthermore, it offers a novel strategy for *ab initio *prediction of alternative splice events.

## Abstract 2

A new mechanism of alternative splicing is proposed which creates a protein feature by putting together two non-consecutive exons and destroys a feature by inserting an exon in its body. Evidence for this rare mechanism is provided by a genome-wide search with four specific protein features.

## Background

Alternative splicing is an important post-transcriptional process and mainly contributes to the complexity of a transcriptome and proteome [1-3]. Alternative splicing often produces two or more proteins with functional differences from one gene [4] but can also downregulate the overall protein level by producing targets for nonsense-mediated mRNA decay [5], which is used, for example, in the autoregulation of splicing factors [6]. Furthermore, defects in splicing are the basis for a number of diseases [7].

One major mechanism of alternative splicing to alter protein function is the insertion/deletion of functional units such as protein domains, transmembrane (TM) helices, signal peptides, or coiled-coil regions. Alternative splicing tends to insert/delete complete functional units instead of affecting parts of a unit [8]. Moreover, several protein domains have a tendency to be spliced out in some transcripts [9,10]. Many proteins occur in a soluble as well as a membrane-bound form. When encoded by a single gene, the soluble form can be produced by post-translational ectodomain shedding [11] or alternative splicing of exons that encode the TM helices. Indeed, 40-50% of the alternatively spliced, single-pass TM proteins have a splice form that specifically removes the TM domain [12,13]. Furthermore, protein forms can differ in their affinity to bind ligands [14,15] or in their subcellular location [16].

In this paper, we present a novel mechanism to modulate function and/or subcellular localization of a protein by alternative splicing. Assuming a protein feature is encoded in two parts by two non-consecutive exons, for example, exon 2 and 4, inclusion of exon 3 results in a protein lacking this feature since it is disconnected at the sequence level. In contrast, the skipping of exon 3 leads to a protein with this feature. We provide evidence for this mechanism by considering four protein features: TM helices, phosphorylation and glycosylation sites, and Pfam domains. In general, this mechanism is conceivable for many other protein features and provides a novel strategy for *ab initio *prediction of alternative splice events.

## Results and discussion

In order to find genes that encode a protein feature by two non-consecutive exons, we searched all human RefSeq transcripts for annotated features that span an exon boundary. For these exon pairs, we searched dbEST to find alternative splice events that insert a sequence between them. Thus, we only selected pairs of exons if they had expressed sequence tag (EST)-confirmed, alternative exons between them that are skipped in the given RefSeq. Apart from alternative exons, intron retention or an alternative donor/acceptor site located in the intron can lead to such an insert. We only selected inserts that preserved the open reading frame. Then we evaluated whether the longer transcript (with the insert) still encodes the feature or not. We only considered two exons for small features like TM helices and post-translational modification contexts since it is unlikely that more than two exons encode the feature. For more complex features like Pfam domains, we allowed for the domain to be encoded by more than two exons.

**Figure 1:**
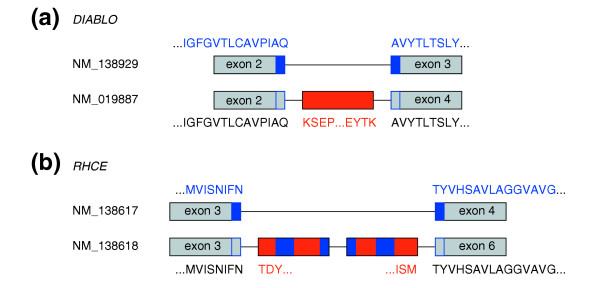
TM domain destruction by exon insertion. **(a) **Exons 2 and 3 of NM_138929 of *DIABLO *encode a TM domain (shown as blue boxes). This TM domain is destroyed in another transcript (NM_019887) that includes an additional exon. The inserted exon (shown in red) encodes many polar amino acids. **(b) **Exons 3 and 4 of NM_138617 of *RHCE *encode a TM domain that is destroyed in NM_138618 by the inclusion of two exons. Interestingly, the two included exons encode three new TM domains. Thus, the skipping of exon 4 and 5 of NM_138618 results in a protein that has only two instead of three TM domains fewer. Exon numbers refer to the respective transcript. TM, transmembrane.

The first protein feature we considered was TM domains. We annotated TM helices in all RefSeq transcripts with the TMHMM program [17]. We found 1,807 TM domains (14% of all TM domains) that are encoded by two exons (Additional data file 1). For ten cases, we found EST evidence for an insert due to alternative splicing. As TM domains are short stretches of hydrophobic amino acids, an insert with polar residues will result in the destruction of the TM helix. Indeed, the evaluation of these ten longer transcripts with TMHMM showed that six clearly lacked the TM domain which, in three cases, leads to a soluble protein (Table 1). An example of the disruption of the single TM domain is depicted for *DIABLO *in Figure 1a. A more complex example is at the Rhesus blood group antigen gene (*RHCE*) where the inclusion of two exons resulted in a loss of one TM domain as well as the gain of three others (Figure 1b). The massive reconstruction of TM domains in the respective protein isoforms can have considerable consequences for the orientation of the proteins within the cellular membrane and for their interaction with other membrane components.

To find further cases of feature disruption by sequence insertion, we applied the procedure to experimentally verified post-translational modification sites. Post-translational modification of proteins plays a role in various important processes. For example, phosphorylation of splicing factors can influence splicing decisions [18] and glycosylation is associated with a modulation of proteolytic resistance and ligand binding [19]. The residue to be modified must be located in a favorable sequence context to be recognized by the enzyme. If this residue is close to an exon boundary, an alternative splice event can change the context to an unfavorable one with the consequence that the modification cannot take place anymore. We inspected the O-GlycBase [19] and Phospho.ELM [20], and found 435 modified residues that are close to 213 different exon-exon junctions. Among them, four exon junctions showed an insert due to alternative splicing. *CCL14 *has a glycosylated serine at position 26, which is the last residue encoded by exon 1. We found two ESTs (AA612866, Z70293) with an included 48-nucleotide exon between exon 1 and 2. The NetOGlyc [21] score for the serine in the new sequence context dropped from 0.97 to 0.35 (threshold 0.5). Thus, the new context might prevent glycosylation of this residue. For *CDK5*, an alternative acceptor (BU529114) that inserts nine amino acids upstream of exon 8 alters the context of the phosphorylated serine at position 159 of the protein. The NetPhos [22] scores of both contexts differ (0.93 vs 0.43, threshold 0.5), which indicates that only one context allows recognition by the kinase and, thus, the phosphorylation of the serine. Additionally, we found two examples (*MGP *and *CDK2*) where an included exon alters the context of a phosphorylated residue, however, the scores for the new contexts dropped only marginally.

**Figure 2:**
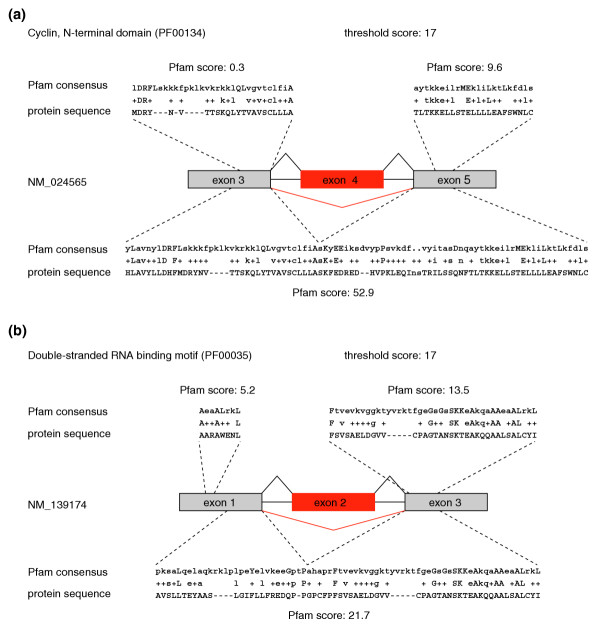
Pfam creation by exon skipping. The alternative exon is shown in red. The two partial Pfam alignments for the RefSeq transcript and the complete alignment for the exon-skipping variant are shown above and below the partial gene structure, respectively. Dashed lines indicate parts of the exon for which a Pfam alignment has been found. **(a) **NM_024585 has a splice form that skips exon 4 (shown in red), which results in the creation of a new domain. The Pfam scores for the separated parts are far below the threshold score of 17 and, thus, the Pfam is not found for the longer transcript. **(b) **Skipping exon 2 of NM_139174 results in a new double-stranded RNA binding Pfam.

For the fourth feature, we considered functional protein domains using the Pfam database [23]. We found 473 inserts into a Pfam domain and nine of those resulted in a disruption of the Pfam (Table 2). Additionally, using the algorithm described in [24], we found three cases where the skipping of a RefSeq exon creates a new Pfam (Table 2). For example, skipping exon 4 of NM_024565 created the cyclin N-terminal domain (Figure 2a). Since exons 5 to 7 of this transcript encode the cyclin C-terminal domain (PF02984), only the exon skipping variant might perform the function of a cyclin. Moreover, skipping exon 2 of NM_139174 resulted in a new double-stranded RNA binding domain (Figure 2b). Downstream of this domain, the transcript encodes an adenosine-deaminase (editase) domain. Thus, the loss of the RNA binding property might act as a negative regulation of the editase activity. Most Pfam domains fold into three-dimensional structures and we cannot rule out that these 12 domains also adopt the correct folding with the insert. However, using standard cut-off scores, these Pfam domains cannot be found in the longer transcripts since the scores for both individual parts are always below the threshold.

In general, any EST-based approach is hampered by the bias of publicly available EST databases towards cancer-related tissues or cell lines that may exhibit aberrant splicing [25,26]. Furthermore, a splice form that is only represented by a single EST may be a rare error by the spliceosome. Therefore, we determined the number and tissue source of the ESTs that match both splice variants for the described examples (Additional data file 2). For seven of the 20 examples, only one splice form is represented by a single EST or by cancer-related ESTs. However, the remaining examples are supported by several ESTs as well as ESTs from normal tissue, and in four cases both splice variants are contained in the RefSeq database. Thus, we conclude that the majority of the described examples are real splice variants and not artifacts or aberrant splice events.

Besides the four features investigated here, there are many others that can only function if they are connected on the sequence level. Such functional sites or motifs often have a linear structure and comprise, for example, signal peptides, post-translational cleavage sites and subcellular localization signals as well as sites for protein-protein interaction. Many of these motifs are collected in the Eukaryotic Linear Motif (ELM) database [27]. Such features can lose their function if an insert separates them on the sequence level. For example, splicing at an alternative donor site of the protein kinase C delta leads to an insert of 26 amino acids into a caspase-3 cleavage site and to an isoform that is caspase-insensitive [28]. We have not investigated such features here since only a fraction of them have been experimentally verified and a prediction results in a high number of false positives. With further efforts in verifying and characterizing these features, we expect an increasing number of examples for the proposed mechanism of modulating protein function by alternative splicing. Interestingly, the same principle was recently used to experimentally characterize exon splicing silencers (ESS) [29]. In this study, ESS candidates were inserted in the middle exon of a three-exon minigene. If a candidate ESS acts as a silencer, the middle exon is skipped and only in this case a functional green fluorescent protein is encoded. Furthermore, this mechanism is not restricted to protein features but it is also conceivable for sequence and structural features at the mRNA level. For example, some of the variable first exons of *NOS1 *together with exon 2, form a hairpin structure that is involved in translational regulation, whereas other alternative first exons do not allow hairpin formation [30].

From an evolutionary viewpoint, this mechanism can be explained in two ways depending on whether the protein feature is ancestral or not. If the feature is ancestral, it means it is initially encoded by two neighboring exons and the inserted part must have appeared in the intronic sequences [31-33]. In this case, the insert simply has the function of a spacer. If the feature is not ancestral, it means the longer splice form is evolutionarily older and, therefore, the alternative exon or splice site must have been converted from a constitutive to an alternative one. This can happen, for example, by the weakening of splice sites or the creation of ESS [34]. Complex features with a high sequence specificity such as Pfam domains are likely to be ancestral. In contrast, small features with a loose sequence motif such as the context of a post-translational modification site can arise just by chance and can therefore be evolutionarily younger.

Not all alternative splice events are represented in EST databases and, thus, the development of non-EST-based methods for *ab initio *prediction of splice events is a necessary but challenging task. Currently, there is only one method that mainly uses genomic conservation of exons and flanking introns to discriminate between alternative and constitutive exons [35]. Although alternative splicing often deletes functional units, it is very hard to predict such events on the protein level without ESTs. However, a search for protein features that are put together by exon skipping would provide a new way to predict alternative splice events. For that purpose, it has to be assumed that the split feature is unlikely to be encoded by two non-consecutive exons just by chance. Since Pfam domains usually have a high sequence specificity, we tested this assumption for Pfams by skipping 10,962 constitutive exons. We found only four cases (0.036%) where skipping of a constitutive exon results in an additional Pfam domain (Additional data file 3). In contrast, nine of the 473 (1.9%) alternatively spliced inserts into Pfam domains resulted in a loss of the Pfam. The odds ratio of 53 indicates that Pfam domains are unlikely to be encoded by non-consecutive exons just by chance.

## Conclusion

**Figure 3:**
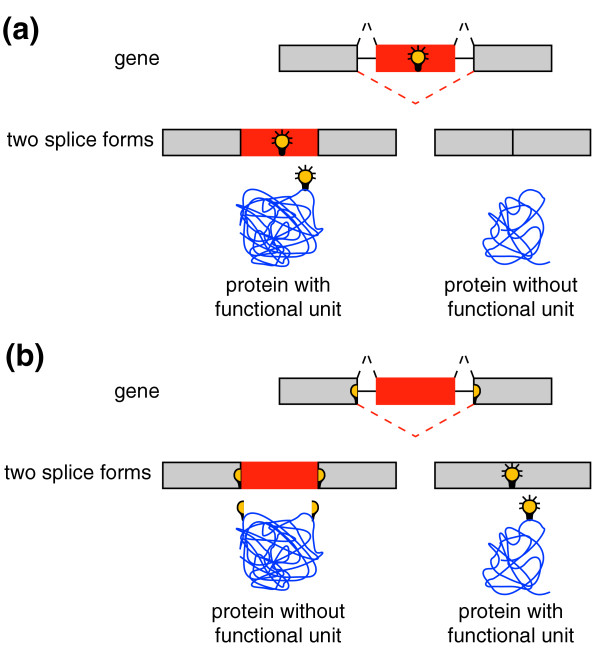
General mechanisms to alter linear protein features by alternative splicing. **(a) **A widespread mechanism is to skip or include an alternative exon (red box) that encodes a functional unit (indicated by the light bulb). The longer splice form with the alternative exon encodes a protein with this feature, the shorter splice form encodes a protein without this feature. **(b) **The novel mechanism involves a functional unit that is encoded by two non-consecutive exons (the two parts of the light bulb). In contrast to the mechanism mentioned above, the longer splice form encodes a protein without the functional unit although both parts are present on the protein sequence. The disruption of the unit results in a loss of function. The shorter splice form encodes a protein that puts together both parts of the unit which results in a gain of function (complete light bulb).

Alternative splicing frequently modulates protein function by insertion or deletion of functional units. In this case, the functional difference is directly associated with the sequence of the inserted or deleted part. Here, we provide evidence for an additional mechanism that acts by putting together a feature from two parts encoded by non-consecutive exons. Thus, the functional difference is not related to a specific insert and the two parts of the feature are present on both the long and the short splice form. The general idea is shown in Figure 3.

Recent alternative splicing databases include the annotation of the functional differences between two protein forms [36]. For this purpose, the novel mechanism described here has to be taken into account since it is obviously not sufficient to inspect the alternative exons in the context of the splice form that includes these exons. The functional difference of the examples shown here can only be found if the complete shorter splice form is investigated simultaneously.

## Materials and methods

### General procedure

All transcripts were taken from the RefSeq annotations in the UCSC Genome Browser (assembly hg16 with annotation March 2004) [37]. For exon pairs that together encode a protein feature, we extracted a 40-nucleotide context (20 nucleotides from the upstream and 20 nucleotides from the downstream exon) and searched, with BLAST, the human fraction of dbEST (August 2004) [38]. We only kept EST hits with two separate HSPs (high-scoring segment pairs).We discarded splice events that resulted in a frameshift and/or introduced a premature termination codon (PTC) since a frameshift leads to a new protein sequence downstream of the alternative splice site and transcripts with PTCs are frequently degraded by nonsense-mediated mRNA decay. Intron retention events were only included if the EST had a spliced intron up- or downstream. For the insertions, we checked presence of AG-GT splice sites. All splice forms were translated with the insertion and a check was made to see if the insert destroyed the feature.

### TM domains

We predicted TM helices with TMHMM for all translated transcripts since, currently, TMHMM was found to be the best-performing TM prediction program [39]. The TM domain location was mapped to the exon structure and we considered a TM helix as encoded by two exons if each exon encoded at least 25% of the domain.

### Glycosylation and phosphorylation contexts

We used Phospho.ELM version 2.0 and O-GlycBase v6.00. The SwissProt IDs were converted to RefSeq IDs with the table from the HUGO gene nomenclature committee website [40]. The location of the modified residues was mapped to the exon structure and we retained those close to an exon boundary (<10 amino acid distance for glycosylated and <5 amino acid distance for phosphorylated residues). To compute the scores for the glycosylated serine, we used NetOGlyc 2.0 because the latest version (3.1) is not able to recognize the serine in the annotated context.

### Pfam domains

Pfam domains were found with hmmpfam using the 'gathering cutoff' scores as given in the Pfam database (version 14). We considered domains with less than 200 residues that are encoded by two or more exons (each exon encodes at least two residues of the Pfam). Additionally, we used the algorithm described in [24] to find cases where the RefSeq transcript is the longer splice form and a shorter exon skipping variant exists that encodes a new Pfam domain. To confirm such candidate splice forms, we searched dbEST with BLAST and the 40-nucleotide context from the up- and downstream exon.

### Test of Pfam domain creation by chance

We compiled a set of 10,962 internal coding exons with a size divisible by three that had at least six ESTs showing their inclusion but no EST indicating their skipping. Those exons were considered to be constitutive. We produced the full-length protein and the shorter protein that corresponds to the hypothetical splice form without such an exon. Then, we used hmmpfam with the gathering cut-offs to search the Pfam database and compared the Pfam family hits for the full-length and the shorter protein.

## Additional data files

The following additional data are available with the online version of this paper. Additional data file 1 is a table listing the TM domains that are encoded by two exons. Additional data file 2 contains the number of ESTs/RefSeqs and information about the tissues or libraries for both splice variants of the examples. Additional data file 3 contains the four cases where skipping of a constitutive exon results in a new Pfam domain.

## Supplementary Material

Additional File 1 TM domains that are encoded by two exons Click here for file

Additional File 2 ESTs/RefSeqs and if available their tissue/library source for the described examples Click here for file

Additional File 3 Pfam creation events by skipping of a constitutive exon Click here for file

## Figures and Tables

**Table 1:** RefSeq transcripts where, due to alternative splicing, sequence insertion destroys a TM helix

Gene symbol	Gene name	RefSeq with TM*	RefSeq/EST without TM^†^	Alternative splice event^‡^	Impact
DIABLO	Diablo homolog (*Drosophila*)	NM_138929	NM_019887	Exon between exon 2 and 3	Disruption of the single TM domain, soluble protein
*DPP8*	Dipeptidylpeptidase 8	NM_017743	NM_197961	Exon between exon 15 and 16	Disruption of the single TM domain, soluble protein
*COX7A2*	Cytochrome c oxidase subunit VIIa polypeptide 2 (liver)	NM_001865	BU570379	Donor downstream of exon 3	Disruption of the single TM domain, soluble protein
*RHCE*	Rhesus blood group, CcEe antigens	NM_138617	NM_138618	Two exons between exon 3 and 4	Disruption of the fifth TM domain, insert contains three new TM domains
na	na	NM_014738	BM693684	Intron between exon 30 and 31	Disruption of the eighth TM domain
na	na	NM_152672	CF147426	Acceptor upstream of exon 4	Disruption of the second TM domain

*RefSeq transcript without the insert (shorter variant) that encodes a TM domain. ^†^Transcript with the insert (longer variant) that destroys a TM helix. ^‡^Exon numbers refer to the RefSeq transcript with the TM helix. na, not approved; TM, transmembrane.

**Table 2:** RefSeq transcripts with an exon skipping splice form that puts together a new Pfam domain

Gene symbol	Gene name	RefSeq/EST with Pfam*	RefSeq/EST without Pfam^†^	Pfam ID	Pfam description	Alternative splice event^‡^	Pfam cutoff score^§^	Score upstream^¶^	Score downstream^¥^	Score combined^#^
na	na	NM_144604	AK056632	PF00642	Zinc finger C-x8-C-x5-C-x3-H type (and similar)	Exon between exon 3 and 4	17.5	-1.2	9.4	23.6
*PRSS25*	protease, serine, 25	NM_145074	AF141306	PF00089	Trypsin	Acceptor upstream of exon 4	23.4	3.0	1.1	30.8
*FOSL2*	FOS-like antigen 2	NM_005253	BX647822	PF00170	bZIP transcription factor	Acceptor upstream of exon 4	23.2	16.1	-4.6	31.3
na	na	NM_003622	AB033056	PF02920	Integrase_DNA	Exon between exon 8 and 9	18.0	13.4	-5.0	21.9
na	na	NM_006832	AK091532	PF00373	FERM domain (Band 4.1 family)	Exon between exon 12 and 13	14.0	-15.9	10.3	15.6
*PQBP1*	Polyglutamine binding protein 1	NM_144494	BM692479	PF00397	WW domain	Acceptor upstream of exon 3	17.0	5.0	9.7	32.5
*MRPL27*	Mitochondrial ribosomal protein L27	NM_148570	BQ028639	PF01016	Ribosomal L27 protein	Acceptor upstream of exon 4	25.0	2.1	8.2	34.0
*PLEKHB1*	Pleckstrin homology domain containing, family B (evectins) member 1	NM_021200	BE703269	PF00169	PH domain	Acceptor upstream of exon 3	22.8	-3.3	11.4	29.7
na	na	NM_020679	BP265352	PF02854	MIF4G domain	Donor downstream of exon 6	14.0	1.1	0.2	17.2
*TRUB2*	TruB pseudouridine (psi) synthase homolog 2 (*E. coli*)	BE793897	NM_015679	PF00849	RNA pseudouridylate synthase	Skip exon 2	14.0	-2.1	-1.3	14.7
na	na	BM903757	NM_024565	PF00134	Cyclin, N-terminal domain	Skip exon 4	17.0	0.3	9.6	52.9
na	na	BC033491	NM_139174	PF00035	Double-stranded RNA binding motif	Skip exon 2	17.0	-5.2	13.5	21.7

*Transcript without the insert (shorter variant) that encodes a Pfam domain. ^†^Transcript with the insert (longer variant) that does not encode a Pfam domain. ^‡^Exon numbers refer to the RefSeq transcript. ^§^Per-domain 'gathering cut-offs' as given in the Pfam database. ^¶^,^¥^Pfam score for the partial domain encoded by the upstream and downstream exon, respectively. ^#^Pfam score for the domain that is encoded by the splice form without the insert. na, not approved.
